# Differential DNA methylation of genes involved in fibrosis progression in non-alcoholic fatty liver disease and alcoholic liver disease

**DOI:** 10.1186/s13148-015-0056-6

**Published:** 2015-03-14

**Authors:** Müjdat Zeybel, Timothy Hardy, Stuart M Robinson, Christopher Fox, Quentin M Anstee, Thomas Ness, Steven Masson, John C Mathers, Jeremy French, Steve White, Jelena Mann

**Affiliations:** Institute of Cellular Medicine, Faculty of Medical Sciences, Newcastle University, 4th Floor William Leech Building, Framlington Place, Newcastle upon Tyne, NE2 4HH UK; Cellular Pathology Department, Royal Victoria Infirmary, Newcastle upon Tyne Hospitals NHS Foundation Trust, Queen Victoria Road, Newcastle upon Tyne, NE1 4LP UK

**Keywords:** NAFLD, ALD, Epigenetics, DNA methylation, Pyrosequencing

## Abstract

**Background:**

Chronic liver injury can lead to the development of liver fibrosis and cirrhosis but only in a minority of patients. Currently, it is not clear which factors determine progression to fibrosis. We investigated whether DNA\methylation profile as determined by pyrosequencing can distinguish patients with mild from those with advanced/severe fibrosis in non-alcoholic liver disease (NAFLD) and alcoholic liver disease (ALD). To this end, paraffin-embedded liver biopsies were collected from patients with biopsy-proven NAFLD or ALD, as well as paraffin-embedded normal liver resections, genomic DNA isolated, bisulfite converted and pyrosequencing assays used to quantify DNA methylation at specific CpGs within *PPARα*, *PPARα*, *TGFβ1*, *Collagen 1A1* and *PDGFα* genes. Furthermore, we assessed the impact of age, gender and anatomical location within the liver on patterns of DNA methylation in the same panel of genes.

**Results:**

DNA methylation at specific CpGs within genes known to affect fibrogenesis distinguishes between patients with mild from those with severe fibrosis in both NAFLD and ALD, although same CpGs are not equally represented in both etiologies. In normal liver, age, gender or anatomical location had no significant impact on DNA methylation patterns in the liver.

**Conclusions:**

DNA methylation status at specific CpGs may be useful as part of a wider set of patient data for predicting progression to liver fibrosis.

## Background

Chronic liver injury of any aetiology can lead to development of liver fibrosis and ultimately cirrhosis [1]. Progression of fibrotic liver disease towards cirrhosis is not linear but rather dynamic and highly variable between individuals. This individual variability is modulated by age, gender and genetic predisposition, which interact with an array of environmental factors such as poor diet, alcohol consumption and smoking to determine progression [2]. Progressive fibrosis and its end stage, cirrhosis, tend to develop very slowly over a period of 20 years or even longer [2]. However, not everyone who suffers chronic liver injury will develop fibrosis or cirrhosis; only a minority of patients reach end-stage liver disease [3,4]. Despite increasing knowledge of the processes underpinning liver disease progression, it is still not possible to predict which patients will progress and which will experience minimal fibrogenesis. This is an important challenge as lack of predictability makes prognosis and patient stratification difficult and limits the rational basis for management options.

Epigenetic processes play a prominent role in a number of complex diseases and may mediate the effects of environmental factors including diet and alcohol [5,6]. This plasticity of epigenetic marks and molecules in response to environmental and genotypic influences may help explain inter-individual differences in responses [7]. Combinations of histone modifications, non-coding RNAs and DNA methylation, in conjunction with transcription factors, ultimately instruct the expression of any given gene in all cells [8]. It is therefore both possible and likely that pre-existing epigenetic marks may at least in part instruct liver disease progression.

DNA methylation occurs at the cytosine base within a cytosine-guanine dinucleotide (often referred to as CpG) where DNA methyltransferase catalyses transfer of a methyl group to the fifth carbon atom within the cytosine ring to form 5-methylcytosine [9]. In many cases, higher levels of DNA methylation around gene promoters correlate with low or no transcription [10]. In humans, the DNA methylation levels at a particular promoter within a given cell type is likely to be very similar; however, there are significant differences in levels of DNA methylation at defined loci between different cell types and tissues. DNA methylation plays an important role in numerous processes, including genomic imprinting, cellular differentiation, embryonic development, X-chromosome inactivation and chromosome stability. Given its importance in regulation of transcription/gene expression and, therefore, in cellular differentiation, errors in DNA methylation impact on multiple disease processes, including cancer [11,12].

Although DNA methylation analysis has not been used to predict liver injury outcome, there have been several studies indicating that changes in locus-specific DNA methylation can affect insulin resistance and severity of non-alcoholic fatty liver disease (NAFLD), as well as predict the response to a low-calorie diet [13-16]. Given these precedents in patient studies, it is conceivable that altered DNA methylation levels may also instruct differential gene expression in development of liver fibrosis. Specifically, we hypothesised that altered patterns of DNA methylation in pro-fibrogenic as well as anti-fibrogenic genes within hepatic cellular compartments in some individuals will impede liver disease progression. In particular, genes known to be involved in specific signalling pathways that enhance the likelihood of progression of chronic liver injury, including *TGFβ1*, *Collagen 1A1* and *PDGFα* may be more highly methylated in individuals that remain fibrosis free, whereas anti-fibrogenic genes such as *PPARα* and *PPARδ* may have higher DNA methylation levels in individuals experiencing fast progress towards severe fibrosis. Ascertaining DNA methylation in patients is possible only via sampling of liver tissue, either by percutaneous liver biopsy, by direct sampling of the organ during liver resection or by sampling of freshly explanted, cirrhotic liver.

Liver biopsy remains the gold standard for assessment of aetiology and fibrosis staging, although there are drawbacks to this method including its inherent invasiveness and significant sampling variability [17]. In this study, we utilise liver biopsy material from mild and severe NAFLD cohorts to assess whether DNA methylation pattern at specific CpGs within pro-fibrogenic and anti-fibrogenic genes can be used as a prognostic indicator of fibrosis progression. Furthermore, we extend these studies into severe ALD, which are compared with normal liver. Finally, we use multiple sampling across the same normal liver to ascertain whether DNA methylation patterns are consistent/stable across the organ and what changes, if any, might be associated with age and gender.

## Results and discussion

### DNA methylation in mild versus severe NAFLD cohort shows significant differences across several CpGs within fibrosis-related genes

We obtained liver tissue from paraffin-embedded percutaneous needle biopsies carried out in eight NAFLD patients with minimal fibrosis and nine NAFLD patients with advanced fibrosis (Tables 1 and 2). The NAFLD cohort was entirely male and the clinical laboratory characteristics, other than ALT and triglycerides, were not significantly different between the two groups (Tables 1 and 2) The difference in age did not reach statistical significance, probably due to lack of statistical power. Histological scoring of all samples was conducted by two expert clinical pathologists. Patients with advanced fibrosis NAFLD exhibited significantly more hepatocyte ballooning and portal inflammation, consistent with the presence of a more active steatohepatitis than those in the mild NAFLD fibrosis group (Table 1).

**Table 1:** **Comparison of the clinical and demographic factors between mild and severe NALFD cohorts**

	**NAFLD (mild fibrosis) F0 to F2 (** ***n*** **= 8)**	**NAFLD (severe fibrosis) F3 to F4 (** ***n*** **= 9)**	**Statistical significance**
Age, mean (95% CI)	51.88 (40.43 to 63.32)	60.56 (54.50 to 66.61)	ns
Male sex (%)	8 (100%)	9 (100%)	ns
AST (IU/L) (95% CI)	45.38 (27.69 to 63.06)	43.86 (32.92 to 54.79)	ns
ALT (IU/L) (95% CI)	79.75 (39.51 to 120)	45.56 (30.5 to 60.96)	*P* = 0.04
BMI (kg/m^2^)	35 (30 to 43.2)	36 (30 to 46)	ns
Cholesterol (mmol/L)	5.2 (3 to 7)	4.0 (2.7 to 5.5)	ns
Triglycerides (mmol/L)	5.8 (2 to 12.5)	2.2 (0.8 to 4.7)	*P* = 0.01
HDL cholesterol (mmol/L)	0.9 (0.6 to 1.2)	1.1 (0.7 to 1.6)	ns
LDL cholesterol (mmol/L)	3.8 (2.1 to 6.2)	2.6 (1.5 to 3.9)	ns
Total/HDL ratio	6.1 (3.3 to 9.5)	3.9 (2.1 to 6.1)	ns
Steatosis			
0 (<5%)	-	1	
1 (5% to 33%)	1	8	
2 (33% to 66%)	5	-	
3 (66%<)	2		
Inflammation			
0	2	1	
1	4	3	
2	2	5	
3			
Ballooning			
0	1	2	
1	7	3	
2	-	4	
Fibrosis			
0	-	-	
1	7	-	
2	2	-	
3	-	4	
4	-	5

**Table 2:** **Comparison of comorbidity data between mild and severe NAFLD cohorts**

	**NAFLD (mild fibrosis) F0 to F2 (** ***n*** **= 8)**	**NAFLD (severe fibrosis) F3 to F4 (** ***n*** **= 9)**	**Statistical significance**
Hypertension (*N*, %)	5 (62.5%)	4 (44%)	ns
Any hypertensive medication (*N*, %)	5 (62.5%)	6 (66%)	ns
T2DM (*N*, %)	8 (100%)	8 (100%)	ns
Insulin treated (*N*, %)	3 (37.5%)	3 (33.3%)	ns
Oral medication treated (*N*, %)	3 (37.5%)	7 (77.7%)	ns
Diet treated (*N*, %)	2 (25%)	1 (11.1%)	ns
Cardiovascular disease (*N*, %)	2 (25%)	2 (22.2%)	ns
Dyslipidemia treated with lipid lowering agents (*N*, %)	4 (50%)	6 (66.6%)	ns
1 Med (*N*, %)	3 (37.5%)	6 (66.6%)	ns
>2 Meds (*N*, %)	1 (12.5%)	0 (0%)	ns

**Figure 1:**
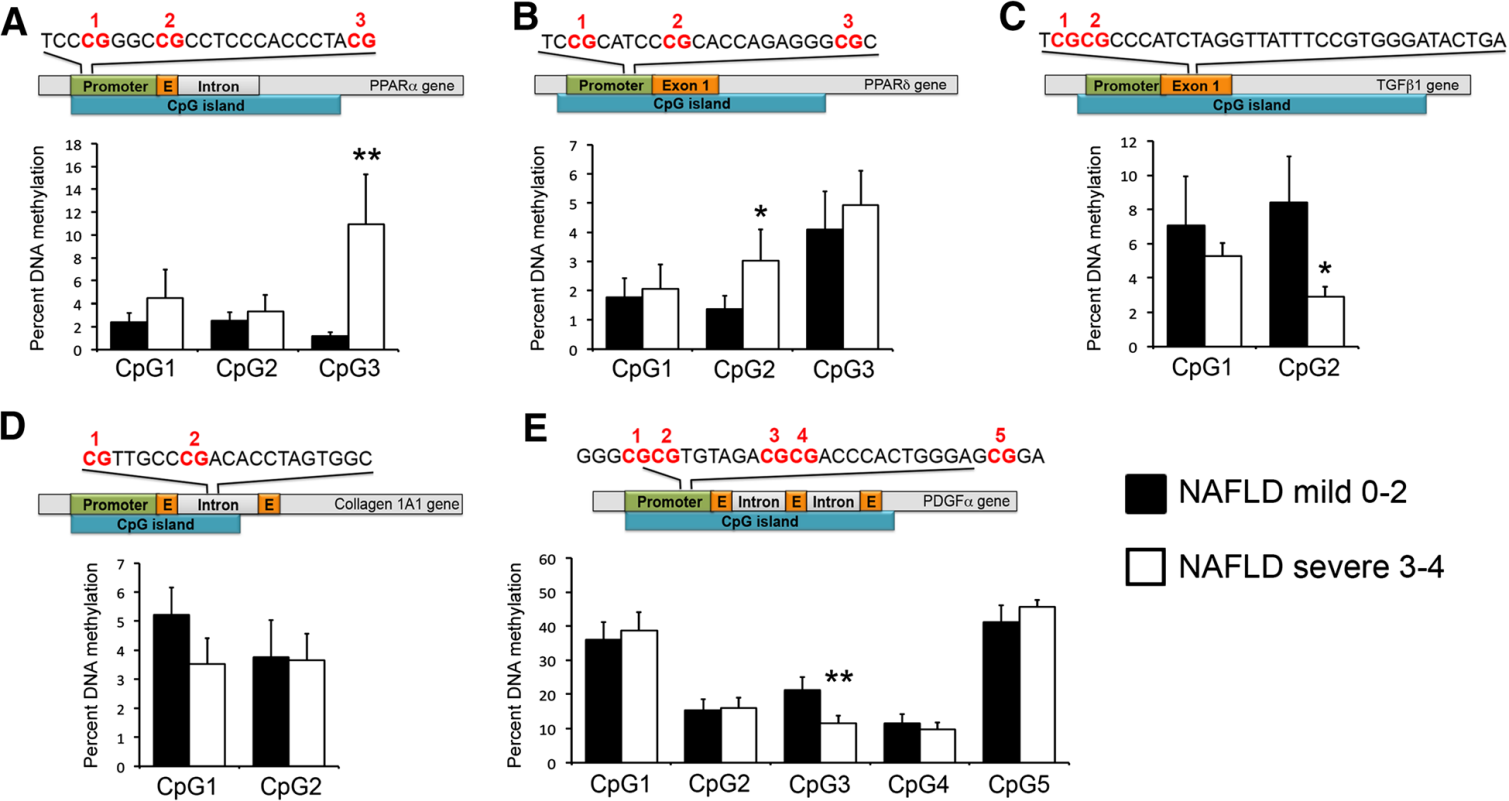
**DNA methylation at particular CG dinucleotides within the human PPARα gene promoter (A), PPARδ gene promoter (B), TGFβ1 exon 1 (C), collagen 1A1 intron 1 (D) and PDGFα gene promoter (E) in liver biopsy tissues from patients with mild (0 to 2 Kleiner score) or severe (3 to 4 Kleiner score) NAFLD was determined by pyrosequencing.** The relative position and surrounding sequence of the differentially methylated CGs are shown in the schematic drawing above the graphs. Differences are expressed as percentage of DNA methylation. Error bars represent mean values ± standard error of the mean (SEM). ^*^ *P* < 0.05; ^**^ *P* < 0.01.

Since higher DNA methylation is associated with gene silencing, we sought to determine whether pro-fibrogenic genes (*TGFβ1*, *Collagen1A1* and *PDGFδ*) are less methylated in severe NAFLD whereas anti-fibrogenic genes (such as *PPARα* and *PPARδ*) might bear higher DNA methylation in the same cohort. To determine if NAFLD-fibrosis severity influences DNA methylation of specific fibrosis-related genes, we isolated genomic DNA from percutaneous needle biopsies in all patients and quantified methylation of specific CpGs within gene regulatory regions of *PPARα*, *PPARδ*, *TGFβ1*, *Collagen1A1* and *PDGFα* as shown in Figure 1. Using pre-validated pyrosequencing assays, we show that out of three CpGs measured, the CpG3 in the target region of the *PPARα* promoter had significantly higher DNA methylation in the severe NAFLD group (10.9% DNA methylation) when compared to the mild NAFLD patients (1.1%, Figure 1A). Similarly, CpG2 within the target region of the *PPARδ* promoter showed statistically higher DNA methylation in the severe group (Figure 1B). However, the opposite effect was observed for the *TGFβ1* gene, where there was lower DNA methylation in those with severe disease (Figure 1C). Although we observed a trend towards lower DNA methylation in CpG1 within the target region of *Collagen1A1*, this was not statistically significant (Figure 1D). Methylation of CpG3 in the *PDGFα* promoter in the severe NAFLD group was only half of that in the mild disease group (11.5% and 21.2%, respectively, Figure 1E). Taken together, these data show that DNA methylation at specific CpGs differs according to fibrosis stage and we hypothesise that this may be linked with alterations in expression of genes known to regulate fibrosis progression. Furthermore, these differences suggest that epigenetic remodelling in liver fibrosis may have clinical relevance.

### DNA methylation in normal human liver versus ALD cohort shows significant differences across several CpGs within fibrosis-related genes

We were next interested to learn whether the differences observed in mild and severe NAFLD were specific to the aetiology of liver disease or due simply to the presence of fibrosis in the liver. To that end, we compared DNA methylation signatures at the CpGs described above in a second liver disease, ALD. Normal human liver had no signs of fibrosis whereas all ALD samples were graded as cirrhotic. The ALD cohort included two samples from end-stage ALD, obtained from freshly explanted organ. The normal human liver cohort (17 patients) had a mean age of 63.3 ± 8.4 years and a 10/7 split of male to female, whereas the ten ALD patients had a younger mean age of 46.3 ± 10.08 years with a 6/4 male to female split (see Table 3). Patients in the normal human liver cohort were taking significantly more anti-hypertensive medication than the ALD cohort (Table 4). The patients were undergoing surgery for major liver resection due to the presence of primary or secondary tumour. Details of resection indication, pre-operative chemotherapy regime, presence of other comorbidities, medication and importantly background liver histology are listed in Table 5.

**Table 3:** **Comparison of the clinical and demographic factors between normal human liver and ALD cohort**

	**Normal human liver (** ***n*** **= 17)**	**ALD (cirrhosis) (** ***n*** **= 10)**	
Age, mean (95% CI)	63.31 (67.94 to 54.71)	46.30 (57.27 to 39.09)	*P* = 0.002
Male sex (%)	10 (58%)	7 (70%)	ns
ALT (IU/L) (95% CI)	-	49 (32 to 69)	
BMI (kg/m^2^)	-	24.2 (19 to 32.5)	
Cholesterol (mmol/L)	-	4.7 (2.0 to 8.7)	
Triglycerides (mmol/L)	-	1.9 (0.9 to 3.6)	
HDL cholesterol (mmol/L)	-	-	
LDL cholesterol (mmol/L)	-	2.9 (1.4 to 5.3)

**Table 4:** **Comparison of comorbidity data between normal human liver and ALD cohort**

	**Normal human liver (** ***n*** **= 17)**	**ALD (cirrhosis) (** ***n*** **= 10)**	**Statistical significance**
Hypertension (*N*, %)	4 (23.5%)	4 (40%)	ns
Any hypertensive medication (*N*, %)	4 (23.5%)	0 (0%)	*P* = 0.046
T2DM (*N*, %)	0 (0%)	0 (0%)	ns
Diet treated	0 (0%)	0 (0%)	ns
Cardiovascular disease (*N*, %)	2 (11.7%)	0 (0%)	ns
Dyslipidemia treated with lipid lowering agents (*N*, %)	3 (17.6%)	0 (0%)	ns
1 Med	3 (17.6%)	0 (0%)	ns
>2 Meds	0 (0%)	0 (0%)	ns

**Table 5:** **Clinical, comorbidity and demographic factors for normal human liver cohort**

**PATIENT**	**Gender**	**Indication resection**	**Pre-op chemo**	**Regimen**	**Comorbidity**	**Diabetes**	**Medication**	**Background liver histology**
1	Female	Intrahepatic cholangiocarcinoma	No		Nil	No	Nil	Bridging fibrosis and chronic cholestasis
2	Male	Colorectal mets	No		Nil	No	Nil	Mild inflammation portal tracts
3	Male	Extrahepatic cholangiocarcinoma	No		Nil	No	Nil	Mild chronic inflammation portal tracts and minimal steatosis
4	Female	Colorectal mets	Yes	FOLFIRI and Bevacizumab	Ischaemic heart disease; hypertension; thoracic outlet syndrome	No	Aspirin; bendroflazide; fluoxetine; isosorbide mononitrate; lansoprazole; simvastatin	Normal
5	Female	Colorectal mets	No		Cleft palate; malnutrition	No	Aspirin; ferrous fumarate	Normal
6	Female	Colorectal mets	Yes	FOLFOX and Cetuximab	Nil	No	Nil	Mild to moderate macrovesicular steatosis; degree of nodular regenerative hyperplasia
7	Male	Colorectal mets	No		Anxiety	No	Amitriptyline; propanolol; tamsulosin, loperamide	Macrovesicular steatosis
8	Male	Colorectal mets	No	FOLFIRI and Bevacizumab	Hypertension	No	Amlodipine; ramipril	Mild steatosis only
9	Female	Colorectal mets	No		Nil	No	Omeprazole; temazepam	Minimal macrovesicular steatosis
10	Female	Colorectal mets	No	Capecitabine and Bevacizumab	Hypertension; hiatus hernia	No	Amlodipine; lansoprazole; pyridoxine; ramipril	Unremarkable
11	Female	Colorectal mets	No		Nil	No	Nil	Unremarkable
12	Male	Colorectal mets	No		Nil	No	Nil	Normal
13	Male	Colorectal mets	No		Nil	No	Nil	Normal
14	Male	HCC	No		Hypertension	No	Atenolol; candesartan; lercanidipine; simvastatin	Moderate macrovesicular steatosis - no fibrosis or steatohepatitis
15	Male	HCC	No		Colorectal cancer	No	Aspirin	Minimal macrovesicular steatosis
16	Male	Colorectal mets	No		Ischaemic heart disease		Ramipril; simvastatin; bisoprolol; aspirin	Mild macrovesicular steatosis and chronic inflammation of portal tracts; no steatohepatitis or fibrosis
17	Male	HCC	No		Breast cancer; Parkinson’s disease; coeliac disease; asthma		Betahistine; Calci-chew; Co-beneldopa; Co-careldopa; ferrous fumarate; fluoxetine; seretide; ipratropium; stalevo; omeprazole; oxytetracyline; ropinirole; salbutamol	Mild sinusoidal dilatation otherwise normal

**Figure 2:**
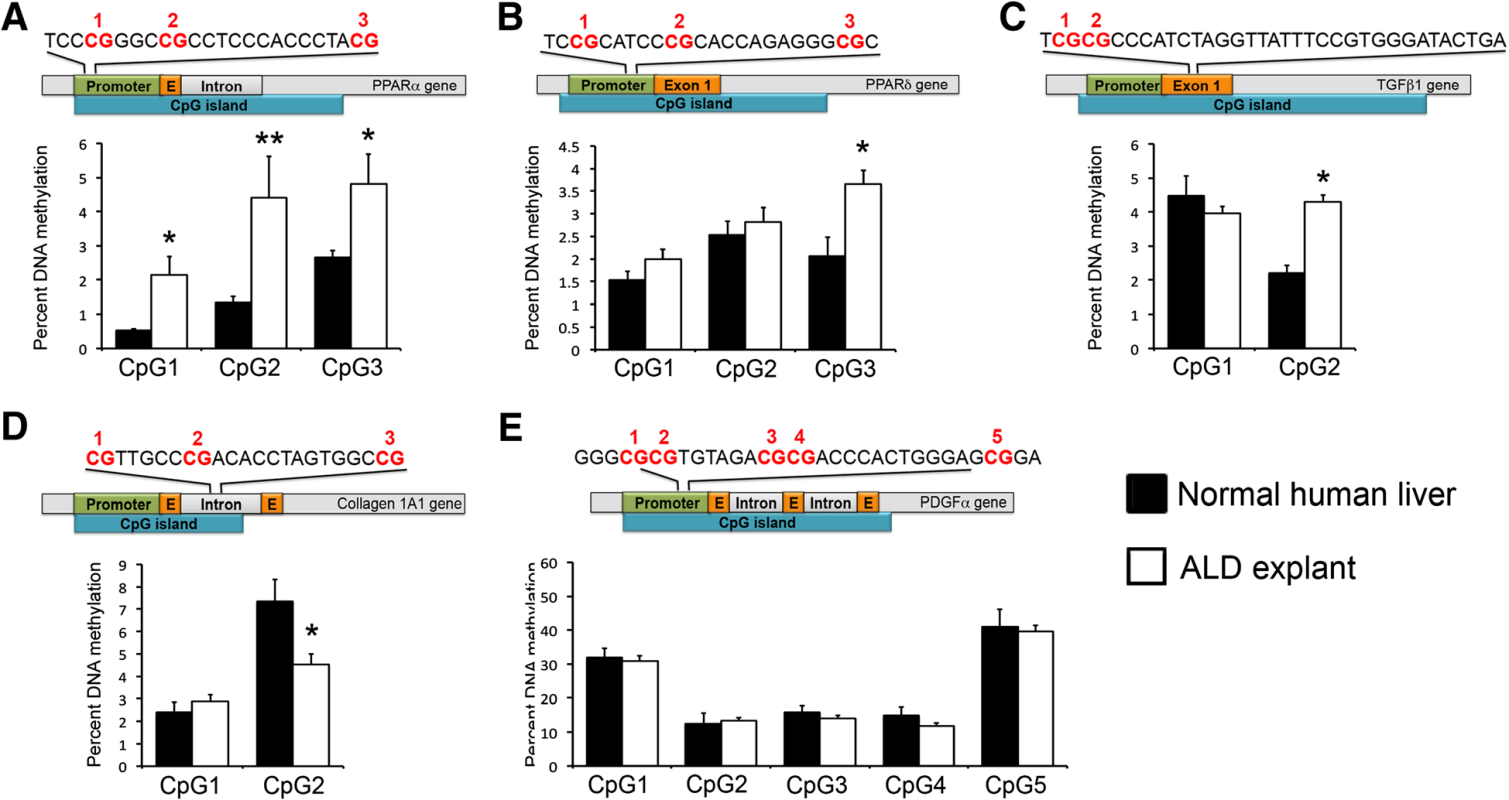
**DNA methylation at particular CG dinucleotides within the human PPARα gene promoter (A), PPARδ gene promoter (B), TGFβ1 exon 1 (C), collagen 1A1 intron 1 (D) and PDGFα gene promoter (E) in resected tissues from normal human liver donors or explanted cirrhotic ALD livers was determined by pyrosequencing.** The relative position and surrounding sequence of the differentially methylated CGs are shown in the schematic drawing above the graphs. Differences are expressed as percentage of DNA methylation. Error bars represent mean values ± standard error of the mean (SEM). ^*^ *P* < 0.05; ^**^ *P* < 0.01.

We observed higher DNA methylation for all three CpGs at the *PPARα* promoter in ALD tissue (Figure 2A). Although these differences were statistically significant, the absolute levels of DNA methylation were overall lower than those in the severe NAFLD tissue (Figures 1A and 2A). In the *PPARδ* promoter, CpG3 in the target sequence had significantly higher methylation in ALD when compared with normal human liver (Figure 2B). Surprisingly, methylation of CpG2 in *TGFβ1* showed increased methylation in the ALD group (Figure 2C). *Collagen1A1* CpG2 was less methylated in ALD (Figure 2D), whereas no methylation differences were detected in any CpGs within *PDGFα* (Figure 2E).

### DNA methylation in normal human liver - age and gender impacts

**Figure 3:**
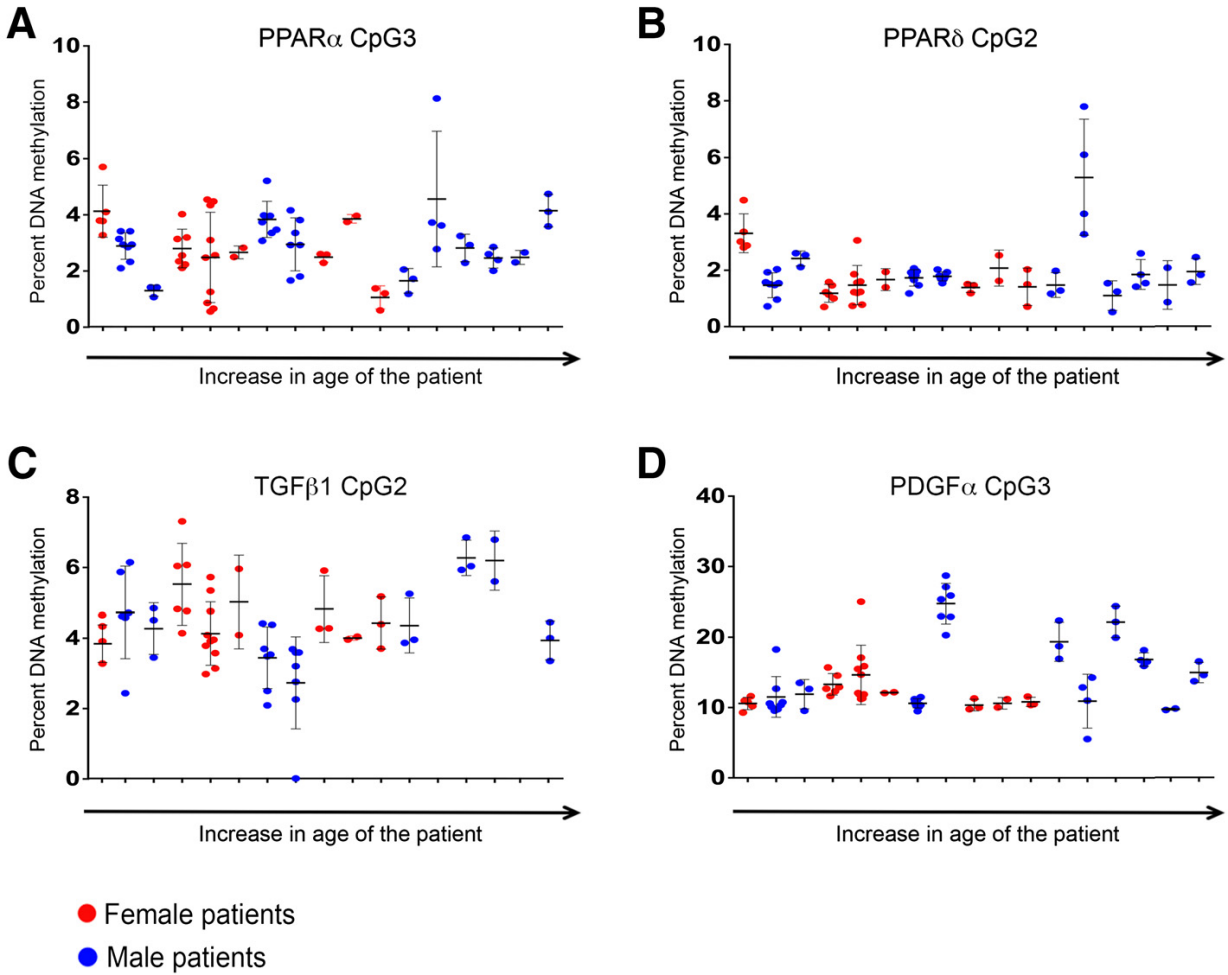
**DNA methylation at CpG3 within the human PPARα gene promoter (A), CpG2 within PPARδ gene promoter (B), CpG2 within TGFβ1 exon 1 (C) and CpG3 within PDGFα gene promoter (D) in a number of samples (ranging from 3 to 9) resected from normal human liver from same donor was determined by pyrosequencing.** Each patient is shown on *x* axis starting from youngest (furthest to the left) then ranked by age up to the oldest on the right of the *x* axis. Female patients are shown in red and males in blue dots. Each dot represents the level of DNA methylation in one sample of donor liver. Results are expressed as percentage of DNA methylation.

A liver biopsy represents around 50,000th of the entire organ, and it is well known that biopsy results can show significant variability of up to 40% for fibrosis staging. In the context of this study, it was important to establish whether single biopsies provided a reliable measure of epigenetic patterns throughout the organ or whether epigenetic signatures differ in different parts of the liver. To this end, we obtained from three to nine samples of normal human liver from 17 patients as outlined in the previous section. The samples were minimally 3 cm and maximally 25 cm apart. We isolated genomic DNA from all samples, carried out bisulphite conversion then quantified methylation at specific CpGs within regulatory regions of *PPARα* (CpG3), *PPARδ* (CpG2), *TGFβ1* (CpG2) and *PDGFα* (CpG3) genes that showed differential methylation in either the NAFLD and/or ALD cohort (Figure 3). Each dot along the same vertical axis represents a unique sample from the same patient (Figure 3A-D). The 17 patients have been ranked according to age from youngest to oldest (Figure 3A-D) with females in red and males in blue. The same patients are also listed in same age order in Table 5, simply labelled 1 through to 17. The results show that, for all target genes, intra-patient variability in DNA methylation was relatively low for samples taken a various locations within the organ. For example, DNA methylation at *PPARα* CpG3 shows around 2% intra-patient/ intra-hepatic variability (Figure 3A) with similar variability for *PPARδ* CpG2 (Figure 3B) and *TGFβ1* CpG2 (Figure 3C) while *PDGFα* CpG3 has a slightly higher intra-patient/intra-hepatic variability of just under 5% (Figure 3D). In summary, neither age nor gender appeared to affect DNA methylation patterns in the selected gene panel in human liver.

## Conclusions

We report a targeted DNA methylation study in which we quantified methylation of specific CpGs at defined loci across five genes involved in the regulation of fibrogenesis. Our cohorts consisted of NAFLD patients with mild and advanced fibrosis, cirrhotic ALD patients and normal human livers. The results were generated from pyrosequencing assays carried out on genomic DNA isolated from percutaneous liver biopsy material in all of the NAFLD and eight ALD patients, with the remainder of ALD and all normal human liver samples being collected during transplantation and surgical resection, respectively.

Approximately half of all human gene promoters have dense clustering of CpG dinucleotides, known as CpG islands. As a general rule, hypermethylation of CpG islands is associated with gene repression, whereas hypomethylation is permissive to transcription [18]. As such, we hypothesised that genes known to drive fibrogenesis, including *TGFβ1*, *Collagen 1A1* and *PDGFα*, might be more highly methylated and consequently be expressed at a lower level in patients that do not progress to severe fibrosis but rather retain a mild phenotype despite on-going liver injury. Conversely, anti-fibrogenic genes *PPARα* and *PPARδ* would be expected to have less DNA methylation in the non-progressor/normal human liver group. In confirmation of our hypothesis, we show that specific CpGs within *TGFβ1* (CpG2) and *PDGFα* (CpG3) (Figure 1B,E) are significantly more methylated in patients with mild fibrosis whereas *PPARα* (CpG3) and *PPARδ* (CpG2) show considerably less methylation in the same group (Figure 1A,B). We next compared cirrhotic ALD livers with normal livers and found that the anti-fibrogenic genes *PPARα* (all CpGs) and *PPARδ* (CpG3) had less methylated DNA in normal livers, which is in line with the results obtained from mild NAFLD. This poses the interesting possibility that the methylation status of a panel of genes may predict predisposition towards development of liver fibrosis irrespective of the liver injury, a very important factor in disease progression that is currently impossible to predict. If a sufficient number of genes harbour differential DNA methylation at particular CpGs, it may be possible to generate an algorithm that can predict which patients are likely to progress on to a severe phenotype versus those that will remain fibrosis free despite the presence of liver injury. As an example, the combination of higher methylation at *TGFβ1* (CpG2) and *PDGFα* (CpG3) with lower level at *PPARα* (CpG3) and *PPARδ* (CpG2) may be sufficient to stratify a patient into a rapid progressor cohort in need of continued monitoring or specific therapy. However, the numbers of patients included in our current study are not sufficiently large to carry out a high-powered calculation. If it proved possible to stratify patients into progressors and non-progressors on the basis of DNA methylation fingerprint at defined loci, such knowledge could be useful in defining an appropriate clinical trial cohort, for testing of anti-fibrotic drugs, for example. Such a cohort would be more informative because the drug would be tested only in those patients likely to progress onto fibrotic disease with time.

In this study, we have chosen to compare ALD with normal human liver and mild NAFLD with severe NAFLD. To ensure that the chosen cohorts were comparable, ALD livers were always analysed with ‘normal’ livers at the same time and on the same pyrosequencing machine, whereas mild NAFLD samples were analysed with severe NAFLD on a separate machine. The two models, however, were never analysed together at the same time. As such, it is possible that variable factors may exist between the two cohorts that would alter absolute measured values without amending the ratio between the compared patient groups. Hence, the data have been reported separately for ALD and NAFLD cohorts (Figures 1 and 2).

A limitation of our study is that the data have been obtained from cross-sectional analyses. It is possible that DNA methylation status changes with disease progression and that the differences we observe are merely a reflection of the liver fibrosis grade rather than a useful predictive or prognostic measure. This question can only be answered using a cohort of patients with progressive disease in whom serial, longitudinal biopsies are collected. In absence of such longitudinal data, we still have to rely on cumulative knowledge from multiple studies, which suggest that DNA methylation does play a role in liver fibrosis as well as being an underpinning cause of other associated comorbidities and complications.

It is also worth noting that NAFLD patients will have on-going liver injury at the time of biopsy (unless they have experienced substantial weight loss). In contrast, ALD patients’ injury will turn on and off depending on the extent of their drinking and so an additional factor adding variability will be the pattern of alcohol intake, despite the fact that most of the ALD patients used in this study were cirrhotic. Incidentally, these issues will also apply to the patients with ‘normal’ liver tissue; that is, it is not clear whether any of them were occasional to moderate drinkers? Thus, if DNA methylation is influenced by grade of inflammation, then NAFLD patients will have more stable patterns of disease than ALD patients. This would also offer a possible explanation for the inability to truly replicate DNA methylation disease patterns in ALD.

For the first time, we investigated the impact of anatomical location, age and gender on DNA methylation status in the liver (Figure 3). Liver biopsy remains the gold standard for histological assessment of liver disease severity, however, because an individual biopsy represents only a very small part (around one 50,000th) of this complex organ, gene methylation in a single liver biopsy may not provide a good representation of methylation of that gene throughout the organ. Furthermore, it is also not known whether methylation patterns are influenced by gender and age. If they were, this could complicate the use of DNA methylation for diagnostic or prognostic purposes. To answer these questions, we analysed a number of samples taken across distinctly separate regions of the liver, spanning up to 25 cm in distance from the first to the last sample within the same liver. We quantify methylation of specific CpGs within *PPARα* (CpG3), *PPAR*δ (CpG2), TGFβ1 (CpG2) and PDGFα (CpG3), all of which we had shown to be differentially methylated according to disease state (Figures 1 and 2). We observed that intra-individual variation in methylation for each CpG site was quite low and methylation levels did not appear to be affected by age or gender. This suggests that a single liver biopsy may be adequate to provide quantitative estimates of DNA methylation which are representative of the whole organ.

It is important to note that what is termed ‘normal’ human liver in this study clearly is not entirely normal, rather these are normal margins collected from patients undergoing major liver resections to remove primary or secondary tumours. As such, the liver microenvironment may be affected by the presence of tumour or indeed by the treatments patients may have received, ranging from chemotherapy as well as the drugs used to treat comorbidities. Although we cannot exclude the possibility that these treatments may have influenced the methylation pattern, uniformity of the data in Figure 3A-D suggest that this is rather unlikely.

Our study supports previously published work that shows DNA methylation is an important determinant of NAFLD progression in patients [13], as well as of activation of stellate cells and development of fibrosis in animal models [19]. Changes in DNA methylation are reported to accompany steatohepatitis in hepatitis C infection, with such changes preceding HCC emergence [20]. Importantly, previous evidence for interaction of the DNA methylome with cellular phenotype provides a possibility of therapeutic intervention in liver disease. This is based on studies in rats where high methyl-donor diet causes changes to DNA methylation in models of obesity [21], while a methyl donor supplementation to a high-fat-high-sucrose diet is able to reverse progression of liver damage [22]. In addition, offspring of mice that were folate deficient during pregnancy developed greater metabolic derangement (30% higher intrahepatic lipid content) when fed a high-fat diet [23]. Taken together, these studies, along with our current data, provide a rationale for further research into an epigenetic basis of chronic liver disease, which may aid development of better diagnostic and prognostic methods as well as therapeutic intervention.

## Methods

### Human subjects

Use of human tissue was approved by Newcastle and North Tyneside Local Research Ethics Committee (approval number H10/H0906/41). All samples were collected and used, subject to patient’s written consent.

### Study design

Seventeen patients with biopsy-proven NAFLD and ten patients with histologically proven cirrhotic ALD (eight biopsies and two explant materials) from the Freeman Hospital, Newcastle upon Tyne, UK, were included in the study. NAFLD diagnosis was made with abnormal serum transaminases, fatty liver on ultrasound and absence of excess alcohol intake (<30 g/day for males, <20 g/day for females), viral hepatitis (hepatitis B and C and HIV), hereditary hemochromatosis, Wilson’s disease, autoimmune hepatitis, α1 antitrypsin deficiency and drug-induced liver injury. ALD diagnosis was made after the exclusion of other diagnoses as above but including a history of alcohol excess defined as >60 g/day for males and >40 g/day for females. Nineteen non-fibrotic liver tissue samples were obtained from patients who underwent hepatic resections for colorectal cancer liver metastasis. Clinical and laboratory data were collected at the time of biopsy, resection or transplant including basic anthropometrics so that BMI could be calculated. Patients were identified as having type 2 diabetes (T2DM) if they were receiving dietary, oral hypoglycaemic drug or insulin treatment for diabetes or had fasting blood glucose >7.0 mmol/L or glucose >11.1 mmol/L following an oral glucose tolerance test. Percutaneous liver biopsies were performed using a Menghini needle or an 18G BioPince liver biopsy system (Medical Devices Technologies, Gainesville, FL, USA). Liver specimens were assessed by two experienced hepatopathologists. Histological scoring was performed according to the NIH NAFLD Clinical Research Network criteria for NAFLD biopsies [24]. Mild disease was defined as fibrosis stage 0 to stage 2, whilst severe disease was defined as fibrosis stage 3 to stage 4. ALD liver sections were reviewed, classified and staged by an expert clinical pathologist according to criteria published previously [25]. Histological sections were stained with haematoxylin and eosin and sirius red.

### Genomic DNA extraction

DNA was extracted from formalin-fixed paraffin-embedded (FFPE) liver biopsy specimens in the ALD and NAFLD cohort. Two specimens in the ALD cohort were extracted from FFPE cirrhotic explant livers and the rest were needle biopsies. Non-fibrotic liver samples were selected >5 cm away from tumour margin in resected liver tissue. Three curls with the thickness of 10 μm were cut from each paraffin block. FFPE tissues were dewaxed by treating with Clearene (Leica Biosystems, Wetzlar, Germany) and serially dehydrated in 100% then 70% ethanol. DNA was extracted using QIAamp DNA Micro Kit (Qiagen, Germany, catalogue no: 56304). Tissues were lysed at 56°C overnight, treated at 70°C for 30 min to remove crosslinks that were formed by formalin, and the lysate was processed and transferred to spin columns as per manufacturer’s instructions. Genomic DNA was quantified using NanoDrop (Thermo Scientific, Waltham, MA, USA, Nanodrop 2000, UV-vis spectrophotometer).

### Bisulfite modification

Sodium bisulfite conversion was performed using EZ DNA Methylation Gold TM Kit (Zymo Research, Irvine, CA, USA). A 2 μg of genomic DNA was bisulphite modified by incubating at 98°C for 10 min and 64°C for 2 h and 30 min. Product was transferred into columns; desulphonated and washed according to manufacturer’s protocol and eluted in 10 μl of elution buffer. A 2 μl of bisulphite modified genomic DNA (400 ng) was amplified in a PCR mix containing 2 μl of forward and reverse primer, 12.5 μl of HotStarTaq Master Mix Kit (Qiagen, Germany, catalogue no: 203445) and 10.5 μl of water. Amplification of DNA was performed in a thermocycler according to the following PCR conditions: one cycle at 95°C for 6 min, followed by 40 cycles of 95°C for 30 s, annealing temperature of 55°C to 59°C (depending on primer pair) for 30 s and 72°C for 30 s, followed by one cycle at 72°C for 30 s.

### Pyrosequencing

Methylation of specific cytosines within CpG dinucleotides was quantified by pyrosequencing using a Pyromark Q96 MD (Qiagen) instrument. PCR and sequencing primers were obtained from predesigned assays; HsCOL1A101_PM PyroMark CpG assay (Qiagen, PM00065821), HsTGFB101_PM PyroMark CpG assay (Qiagen, PM00073913), HsPPARA01PM PyroMark CpG assay (Qiagen, PM00082635), HsAC147651.101 PM PyroMark CpG assay (Qiagen, PM00031745) and HsPPARD01PM PyroMark CpG assay (Qiagen, PM00121310). A 10 μl of biotin-labelled PCR product was used in each well and combined by streptavidin-coated sepharose beads, washed in 70% ethanol, denatured in 0.01% sodium azide and washed in a wash buffer (Qiagen, PyroMark Wash Buffer, 979008). Sequencing primers were annealed to DNA product at 80°C. Samples were run in duplicate. Assay efficiency was validated by unmethylated and methylated DNA (Qiagen, EpiTect PCR Control DNA Set, 59695). CpG methylation data was analysed by Pyro Q-CpG software 1.0.6. Levels of DNA methylation have been measured independently in the compared cohorts. DNA methylation was measured in ALD livers alongside ‘normal’ livers within the same run using the same pyrosequencing machine to ensure that absolute values of DNA methylation measured were always the same for the specified cohort. DNA methylation in the mild and severe NAFLD samples were also analysed in the same run using a different pyrosequencing machine. The two models were never analysed together at the same time.

### Statistical analysis

Data are expressed as means ± SEM. GraphPad Instat was used to perform Mann-Whitney *U* test or chi-square test where *P <0.05, **P <0.01 or ***P <0.001 were considered significant.

## Abbreviations

NAFLD: non-alcoholic fatty liver disease
ALD: alcoholic liver disease
NHL: normal human liver
PPAR*α*: peroxisome proliferator-activated receptor-*α*
TGF-β1: transforming growth factor beta1
